# Alpha-Fetoprotein and Hepatocellular Carcinoma Immunity

**DOI:** 10.1155/2018/9049252

**Published:** 2018-04-01

**Authors:** Xiaoping Wang, Qiaoxia Wang

**Affiliations:** ^1^Laboratory of Molecular Biology & Pathology, Shaanxi University of Chinese Medicine, Xianyang, Shaanxi 712046, China; ^2^Department of Infectious Diseases, Xi'an Central Hospital, Xi'an, Shaanxi 710000, China

## Abstract

Hepatocarcinoma is one of the most prevalent gastroenterological cancers in the world with less effective therapy. As an oncofetal antigen and diagnostic marker for liver cancer, alpha-fetoprotein (AFP) possesses a variety of biological functions. Except for its diagnosis in liver cancer, AFP has become a target for liver cancer immunotherapy. Although the immunogenicity of AFP is weak and it could induce the immune escapes through inhibiting the function of dendritic cells, natural killer cells, and T lymphocytes, AFP has attracted more attention in liver cancer immunotherapy. By in vitro modification, the immunogenicity and immune response of AFP could be enhanced. AFP-modified immune cell vaccine or peptide vaccine has displayed the specific antitumor immunity against AFP-positive tumor cells and laid a better foundation for the immunotherapy of liver cancer.

## 1. Introduction

Hepatocellular carcinoma (HCC) is one of the most common tumors in the world, with a new incidence of up to 600,000 cases per year [1, 2]. This malignancy is becoming more prevalent in Asia because of the high incidences of hepatitis B and C infections [3, 4]. Due to the insidious onset and long incubation period of HCC, patients usually got intermediate or advanced stage with poor prognosis. At present, the treatments of liver cancer mainly rely on surgical resection, liver transplantation, local ablation, chemoembolization, and molecular targeted therapy, but the effects of these strategies are not ideal and the mortality is still high [5, 6]. Therefore, there is an urgent need to develop effective adjuvant therapies that may prolong the life and improve the quality of patients with HCC.

Immunotherapy of HCC could stimulate the identification of specific tumor antigen, inhibit the proliferation of cancer cells, produce memory lymphocytes and prevent recurrence, so the immunotherapy of HCC has attracted more attention in recent years. In this paper, the research progress on the immunotherapy of alpha-fetoprotein (AFP) and HCC is reviewed.

## 2. Significance of Alpha-Fetoprotein Expression in Hepatocellular Carcinoma

Alpha-fetoprotein is a kind of glycoprotein, derived from embryonic endoderm tissue cells. AFP content in fetal serum is high and gradually decreases to the level of adults after birth. The low content of AFP in the adult blood is mainly due to the loss of the ability to synthesize AFP in mature hepatocytes. When transformed, the liver cancer cells can regain the ability to synthesize AFP. Besides liver cancer, malignant tumors from stomach, pancreas, and reproductive system are often accompanied by a small amount of increased AFP. In general, AFP is produced only at low levels after birth; however, majority of human HCC overexpress high level of AFP in eastern populations [6, 7]. Therefore, the HCC patients with AFP negative or weak positive in serum is usually consistent with the characteristics of high differentiated cancers [6, 7].

Combining detection of AFP with new diagnostic markers increases the sensitivity and specificity to diagnose HCC. For instance, combination of AFP and lens culinaris lectin detection resulted in increased specificity and sensitivity in diagnosing HCC [8, 9]. With the progress of clinical laboratory techniques, the new liver biomarkers have displayed important roles in the diagnosis of liver cancer. Abnormal prothrombin des-*γ*-carboxy prothrombin (DCP) and alpha-L-fucosidase (AFU) are available in diagnosing AFP-negative HCC [10, 11]. Combining AFP-L3 with Golgi protein 73, the diagnostic sensitivity was 94%, specificity was 93.1%, and the accuracy rate of diagnosis was 93.3% in HCC patients, greatly improving the early diagnosis rate of liver cancer [12, 13].

In addition to being an oncofetal antigen and diagnostic marker for liver cancer, AFP possesses a variety of biological functions, such as transport function similar to the class of albumin family, transporting metal ions, drugs, bilirubin, and steroids [1–4].

## 3. AFP and Immune Escape Mechanism of Hepatocellular Carcinoma

Liver cancer cells can modify their own surface antigen and change the microenvironment around the tumor lesion to implement their immune escape. Exogenous AFP can not only promote the proliferation of hepatocellular carcinoma cells and the formation of tumor blood vessels, but also enhance the antiapoptosis effect of cancer cells [14–18]. Thus, AFP plays an important role in the development and progression of liver cancer. Cellular immunity is the main immune mechanism of anticancer [19]. Dendritic cells, natural killer cells, and T lymphocytes are involved in immune surveillance [19]. It is confirmed that AFP could affect the three important immune cells to exert their antitumor effects [14, 15].

DC cells own the function of high uptake, processing and presenting antigen, which are the only antigen-presenting cells (APC) directly activating naive T cells. AFP can inhibit the maturation and induce apoptosis of DC cells, so that cancer cells could escape the immune surveillance. Researches demonstrated that when peripheral blood mononuclear cells were cultured in normal umbilical cord blood AFP (nAFP) and tumor derived AFP (tAFP) separately, the phenotype and function of DC cells were altered. Tumor derived AFP could significantly inhibit the differentiation of DCs, and the DC cells restricted by tAFP still maintained the immature mononuclear cell-like morphology [20]. Moreover, the DCs apoptosis index and Caspase-3 expression were significantly increased after AFP treatment [20, 21]. In addition to the increased expression of Caspase-3, p38 mitogen-activated protein kinase (MARK) expression was also increased in DC cells [20, 21]. When AFP was incubated with DCs from human peripheral blood, the cells were found to express Caspase-3 and p38-MARK, which would induce apoptosis and inhibit the maturation of DC cells, thus resulting in the immune escape of cancer cells [21, 22].

NK cell is an important component of the innate immune system, which is the first line of defense against infections and tumors. NK cells could express a variety of surface receptors, including activating receptors (NKG2D, NKp30, NKp44, NKp46) and inhibitory receptors (KIR2DL3/CD158b). When recognizing the tumor cells, the expression of activating receptors increases and the expression of inhibitory receptors decreases, which lead to the enhancement of the antitumor effects. IL-12 is able to stimulate the expression of NKG-2D on the surface of NK cells and improve the toxic effect of NK cells. After binding to its ligand, NKG2D activates NK cells and exerts an immune response [23]. Therefore, when the mice loaded with liver cancer cells were vaccinated with IL-12, it was found that the expression of activating receptors of NK cells increased while inhibitory receptors decreased, and IFN-*γ* content elevated, thus suppressing the tumor growth [22]. The function of NK cells could also be affected by AFP. AFP does not directly damage the function of NK cells, but through inhibiting the maturation of DC cells, DC cells reduce the secretion of the amount of IL-12, then indirectly inhibiting NK cells [24, 25].

**Figure 1:**
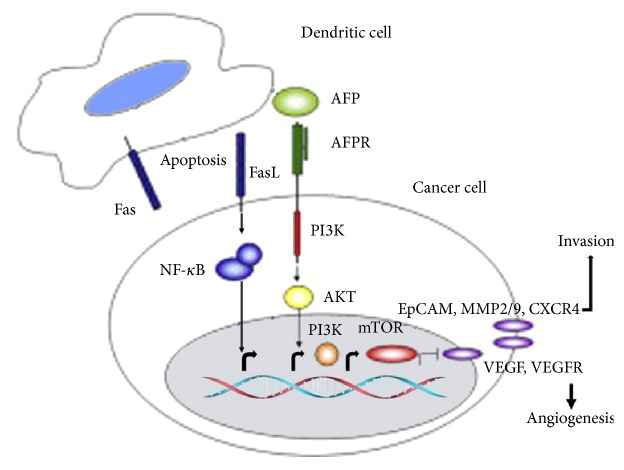
*Mechanisms of AFP promoting the growth of cancer cells*. AFP promoted cancer cell proliferation, invasion, and metastasis by inducing apoptosis of immune cells, binding AFP receptors (AFPR), and activating signal transduction pathways.

DCs could activate AFP-specific T cells significantly in vitro and in vivo. The main mechanism is the specific upregulation of IL-2, IFN-*γ*, TNF-*α*, granzyme, and perforin and downregulation of IL-10, which suppresses the immune function [34]. CD4^+^ T cells as T helper lymphocytes can assist CD8^+^ cytotoxic T cells (CTLs) to exert cytotoxic effect. AFP-specific CD4^+^ T and CD8^+^ T cells kill the cancer cells mainly relying on the granzyme and perforin pathways without affecting the Fas/Fasl pathways [25, 34]. Although AFP is expected to play a certain role in antitumor immunity, it is still useless to stimulate the immune cells and produce substantial effects on cancer cells due to its weak immunogenicity. On the contrary, AFP contributes to the promotion of the change of CD4^+^ T and CD8^+^ T cell proportion and leads to tumor immune escape [33, 35]. Researchers measured CD4^+^ T (Treg cells) in HCC patients, chronic hepatitis B patients, and healthy population and found that the quantity of Treg cells was high in the peripheral blood of HCC patients, which could inhibit antitumor immune responses and affect the function of CD8^+^ T lymphocytes [33, 35]. By multiple ways suppressing the immune cells, HCC cells are able to escape immune surveillance and continue to grow in the body, showed as Figure 1, Box 1.

## 4. Role of AFP in Hepatocellular Carcinoma Immunity

AFP plays an important role in the regulation of cell proliferation, which may be mediated by receptor, signal transportation, and gene expression. Some studies have indicated that AFP is mainly present on the cell surface or inside the cytoplasm, and AFP in the cytoplasm is mediated by the endocytosis of the receptor into the cell [36]. In the AFP-positive tissue, AFP receptor (AFPR) is also positive, while in normal tissue or AFP-negative tissue, AFPR is also negative, indicating that the expression of AFP receptors in the cells is associated with AFP [36]. The promotion of tumor proliferation of AFP/AFPR is dependent on the CAMP-PKA pathway and the induction of Ca^2+^ influx [37]. AFP/AFPR brings about Ca^2+^ influx, so the intracellular Ca^2+^ increases, then the intracellular CAMP correspondingly rises, enhancing protease A activity, prompting DNA synthesis, and achieving the purpose of tumor cell proliferation [37].

The activation of immune response requires enhancing the ability of antigen-presenting cell uptake, processing and presenting antigen, so that T lymphocytes could be activated and the immune response is elicited [38]. Since AFP promotes the proliferation of liver cancer, it could be a new target for the immune therapy of liver cancer. However, despite exposure to high plasma levels of AFP during hepatocellular carcinoma development, only low immunity is mounted against the protein [36, 37]. How to overcome the immune tolerance of AFP is the key point of antitumor immunity. Studies have dissected the immunodominant epitopes of AFP in order to effectively use this protein for immunotherapy of AFP expressing tumors [39–41]. Researches confirmed that these identified epitope peptides could be recognized by the T cells* in vitro *and generated AFP-specific CTLs [39, 40]. Some epitopes of AFP have been found to be recognized by the major histocompatibility complex (MHC) class I or II molecular of APC cells [41]. When presented to CD8^+^ T or CD4^+^ T lymphocytes, the epitopes activated specific CTLs to secret high levels of TNF-*α* and IFN-*γ* and stimulated specific immune response against hepatocellular carcinoma [41, 42]. DC cells modified by the hAFP peptide (137–145) could successfully induce the peripheral blood mononuclear cells to be specific CTLs, and the immune response was better than that of the intact hAFP alone [43, 44]. Thus, AFP could be a promising candidate for immunotherapy. In this way, we may overcome the immune tolerance of AFP and eliminate the cancer cells.

Tumor immunity has the advantages of high efficiency and low toxicity, whose purpose is to start or restart the immune response independently and continuously and avoid the damage of autologous tissue cells. At present, clinical studies have confirmed that immunotherapy can improve the prognosis of patients with HCC [43]. It was found that when DC cells were incubated with AFP and IL-12, specific T lymphocytes were activated, resulting in a large number of IL-12 and TNF produced, and specifically attacking HCC cells. The study indicated that DC cells activated by AFP could be made for DC vaccine to induce specific immune effect [45–48]. Research found that the combination of the “internal immune adjuvant” heat shock protein 70 (HSP70) with AFP, forming a HSP70-AFP complex, enhanced AFP immunogenicity and stimulated the immune system to exert its effect [49, 50]. Heat shock protein has the property of “molecular chaperone,” which can help the protein fold, transportation, repair, and degrade. Heat shock protein 70 can present antigen, activate the immune cells, and promote the transformation of Th0 cells to Th1. The tumor HSP70 may combine with a variety of tumor antigen peptides and present multiple antigen epitopes to T cells. HSP70 could activate stronger DC cells response and gain a large amount of cytokine release from the polyclonal T cells [51–53]. Studies have verified that the C-terminal of the HSP70 has immunogenicity and can stimulate the immune cells to produce chemokines, which is able to induce DC mature [51, 52]. The N-terminal of HSP70 plays an important role in presenting antigen peptide. The HSP70 presents antigen to APC cells by HSP70-peptide complexes, then activating CTLs by the interaction with MHC-class I molecules, promoting DC cells and macrophages to secrete inflammatory cytokines, upregulating CD8^+^ T cells and IFN-*γ* content, and lowering the concentration of TGF-*β*1 [52].

## 5. Application of AFP in the Immunotherapy of Hepatocellular Carcinoma

**Figure 2:**
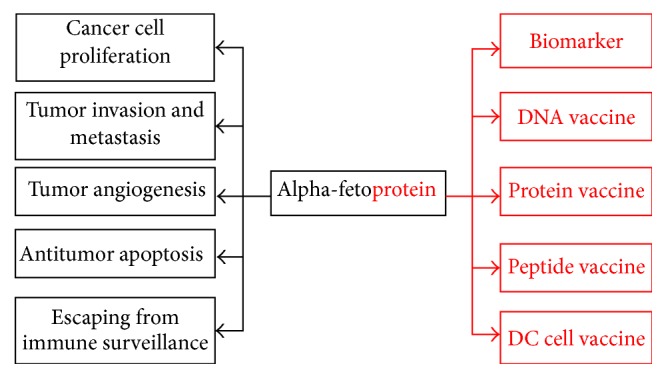
*AFP-a sword with two blades*. As an oncofetal antigen and diagnostic marker for liver cancer, AFP is a sword with two blades. AFP promoted tumor proliferation, invasion, angiogenesis, and escaping from immune surveillance, which could be an ideal target for immunotherapy. On the other side, AFP-modified DNA, protein, peptide vaccine, or DC cell vaccine displayed the specific antitumor immunity against AFP-positive liver cancers.

Liver cancer vaccine aims to make use of HCC cells or antigen to induce the specific immune response, achieving the purpose of prevention and treatment of liver cancer. AFP as a tumor-associated antigen can promote the development of cancer and affect the expression of oncogene through the change of cAMP and Ca^2+^ concentration via binding with the cell membrane receptor [37]. Researches indicated that blocking AFP could inhibit the proliferation of hepatoma cells and induce apoptosis of cancer cells [54–56]. Therefore, AFP has become a new target for the treatment of liver cancer (Figure 2, Box 2).

Recently, there has been an increase in the development of therapeutic vaccines, which are expected to target specific tumor-antigens and boost the host's immune system. Thus, therapeutic vaccines can be considered for the treatment of HCC because about 70% of HCC patients express high levels of the alpha-fetoprotein (AFP), which can serve as a target for immunotherapy [3, 4]. However, because of the suppressing of cellular immunity and the immunologic tolerance of AFP, current treatments targeting AFP are generally weak and do not provide reliable antitumor protection [5]. A number of therapeutic approaches have been described to improve preexisting antitumor immunity, including recombinant plasmid DNA, chimeric virus-like particles, viral or bacterial vectors expressing AFP proteins, and adoptive transfer of tumor-specific T cells [57–62]. However, therapeutic vaccination is limited due to the low response of the immune system to AFP antigen. Therefore, there is a need to boost the host's immune response against AFP by improving the immunogenicity of AFP.

Polypeptide vaccine has the characteristics of immune response, safety, and easy production. At present, antivirus, anticancer, antimalaria, contraception, and other peptide vaccines are under clinical trials [62, 69–72, 74, 75]. In the immunotherapy of HCC, DC vaccine has showed good application prospects because of its specific and effective immune response in vivo. Current researches have focused on how to enhance the antigen-presenting function of DC cells and exert its strong ability to kill HCC cells [45–48]. Researches showed that DC cells stimulated with zoledronic acid could make T cells secrete large amounts of interferon and enhance the cytotoxic effects on CTLs [72]; DC cells separated from the patients with HCC were cocultured with IL-2 and GM-CSF for 2 days, and then implanting the DC cells through arterial catheter to the patients could make the serum produce a large number of IL-9, IL-15, and TNF, prolonging life in patients with HCC [69, 70, 72]. Clinical observation suggested that DC cells loaded with AFP showed good safety and tolerability for patients [69, 70]. The ability of CTLs induced by AFP antigen epitope polypeptide vaccine was stronger than that of oligopeptide vaccine, specially attacking AFP-positive tumor cells [71]. Recently, research found that DCs produced high levels of IFN-*γ* and TNF-*α* when pulsed with tumor-associated antigens (TAAs) and the percentage of regulatory T cells (Tregs) decreased in patients after DC-CTLs therapy [73], which may be a strategy to elicit effective immune response by combining AFP with other TAAs. Due to the weak immunogenicity, heat shock protein as a kind of “molecular chaperone” and “immune adjuvant” has attracted more attention. Most liver cancer cells express a high level of AFP and HSPs in the cytoplasm and HSPs could transport cytoplasm AFP to the cell membrane and release AFP antigen to serum [71, 72], which indicates that AFP could be an ideal target for immune attack. In a previous study, we introduced HSP70 and several AFP peptides into a eukaryotic expression vector and then injected the recombinant vector into mice. As a result, the recombinant vaccine encoding AFP and HSP70 produced effective AFP-specific T cell response and effectively suppressed the growth of tumor cells, eliciting robust protective immunity against AFP-positive tumors in vivo [73]. Consistent with the findings further researches have also confirmed that conjugating HSP70 to AFP antigen could improve the potency of AFP fusion vaccines [49, 50, 63–68]. In recent years, we developed a series of potential therapeutic proteins or peptide vaccines HSP70-AFP, HSP70-AFP-P, and gp96-AFP-P; the recombinant vaccine can induce specific antitumor immunity against AFP-positive tumor cells [49, 50, 67]. Most of all, a novel therapeutic vaccine, HSP70-P/AFP-P, was constructed to enhance the immunogenicity of AFP by conjugating AFP epitope peptide with the HSP70 functional peptide via peptide synthesis. The results verified that vaccination of mice with the novel HSP70-P/AFP-P vaccine mounted strong natural killer cell and CD8^+^ T cell responses, inducing a protective effect against AFP-producing tumors in mice [68]. A systemic meta-analysis indicated that cellular immunotherapy was a feasible adjuvant treatment that could be beneficial for the improvement of short-term response and long-term survival of HCC patients after minimally invasive treatment [76]. Recently, clinical trials showed that nivolumab, a programmed cell death protein-1 (PD-1) immune checkpoint inhibitor, produced durable objective responses for treatment of HCC, indicating the possibility of combining nivolumab with AFP to suppress the proliferation of cancer cells [77]. However, the potential to revert an advanced HCC is a long way, which makes immunotherapy a very attractive adjuvant therapeutic option to be combined with other established treatments such as resection, transplantation, TACE, sorafenib/regorafenib, or nivolumab for the therapeutic design.

## 6. Summary

AFP plays an important role in the development of hepatocellular carcinoma, which can serve as a target for immunotherapy of HCC. Although the current application of immunotherapy for HCC is still on trial, immunotherapy has achieved certain effect in the treatment of liver cancer. With the rapid progress of research, immunotherapy as an important strategy is expected to bring a great prospect for HCC in the future.

## Figures and Tables

**Box 1:**
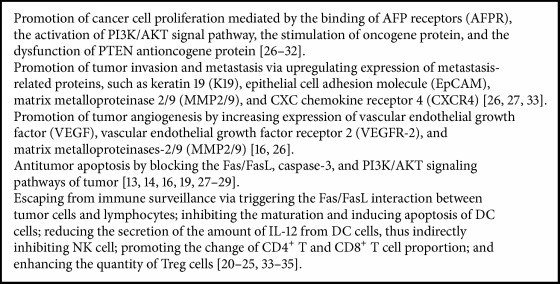
The mechanisms of AFP promoting cancer cell proliferation.

**Box 2:**
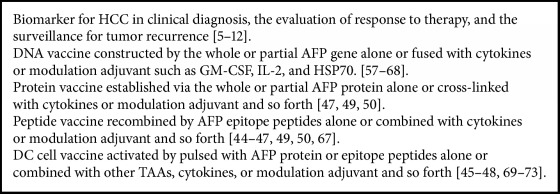
AFP and tumor immunity.
